# Calcium-Dependent Calpain Activation-Mediated Mitochondrial Dysfunction and Oxidative Stress Are Required for Cytotoxicity of Epinecidin-1 in Human Synovial Sarcoma SW982 Cells

**DOI:** 10.3390/ijms21062109

**Published:** 2020-03-19

**Authors:** Bor-Chyuan Su, Chao-Chin Li, Jiun-Lin Horng, Jyh-Yih Chen

**Affiliations:** 1 Department of Anatomy and Cell Biology, School of Medicine, College of Medicine, Taipei Medical University, Taipei 110301, Taiwan; subc8265@tmu.edu.tw (B.-C.S.); jlhorng@tmu.edu.tw (J.-L.H.); 2 Institute of Cellular and Organismic Biology, Academia Sinica, Taipei 115201, Taiwan; jasper15@gate.sinica.edu.tw; 3 Marine Research Station, Institute of Cellular and Organismic Biology, Academia Sinica, 23-10 Dahuen Road, Jiaushi, Ilan 262204, Taiwan; 4 The iEGG and Animal Biotechnology Center, National Chung Hsing University, Taichung 402204, Taiwan

**Keywords:** epinecidin-1, synovial sarcoma, calcium, calpain, oxidative stress, mitochondrial dysfunction

## Abstract

Synovial sarcoma is a rare but highly malignant and metastatic disease. Despite its relative sensitivity to chemotherapies, the high recurrence and low 5-year survival rate for this disease suggest that new effective therapeutic agents are urgently needed. Marine antimicrobial peptide epinecidin-1 (epi-1), which was identified from orange-spotted grouper (*Epinephelus coioides*), exhibits multiple biological effects, including bactericidal, immunomodulatory, and anticancer activities. However, the cytotoxic effects and mechanisms of epi-1 on human synovial sarcoma cells are still unclear. In this study, we report that epi-1 exhibits prominent antisynovial sarcoma activity in vitro and in a human SW982 synovial sarcoma xenograft model. Furthermore, we determined that calcium overload-induced calpain activation and subsequent oxidative stress and mitochondrial dysfunction are required for epi-1-mediated cytotoxicity. Interestingly, reactive oxygen species (ROS)-mediated activation of extracellular signal-regulated kinase (ERK) plays a protective role against epi-1-induced cytotoxicity. Our results provide insight into the molecular mechanisms underlying epi-1-induced cell death in human SW982 cells.

## 1. Introduction

Synovial sarcoma is a rare, malignant, soft tissue sarcoma that is characterized by the fusion of SS18 with any of several SSX genes, including SS18:SSX1, SS18:SSX2, and SS18:SSX4 [1,2]. The disease comprises approximately 8–10% of soft tissue sarcomas [2]. Compared to other types of cancers, synovial sarcomas are relatively susceptible to chemotherapies [2]. Despite this susceptibility, synovial sarcoma has a poor prognosis; the 5-year survival is less than 40%, and about 50% of patients suffer metastatic recurrence within 10 years [1]. Currently, standard therapeutic approaches for local tumors include surgical excision combined with radiotherapy and/or (neo)adjuvant chemotherapy. Anthracycline-based regimes are the first-line therapy [3,4]. Second-line chemotherapeutic agents for synovial sarcoma include Pazopanib, Trabectedin, and Eribulin [4,5,6,7]. For metastatic cases, chemotherapy is the main treatment [2]. However, the combined conventional chemotherapeutic agents gemcitabine and docetaxel do not further improve treatment outcomes in metastatic synovial sarcoma patients [8]. Because of the poor treatment outcomes and low 5-year survival rate, new therapeutic options are needed for this disease.

Epinecidin-1 (epi-1) is a marine antimicrobial peptide identified from orange-spotted grouper (*Epinephelus coioides*), which exhibits broad-spectrum bactericidal [9], antiparasitic, and immunomodulatory activities [9,10,11,12]. Circular dichroism spectroscopy analysis revealed that epi-1 folds into an α-helical structure when it binds to a membrane-like environment [13], and this structure may be essential for its cytotoxicity to microbes. In addition, epi-1 possesses anticancer activity in human fibrosarcoma, human leukemia cancer cells, and glioblastoma cells [13,14]. However, its effects on human synovial sarcoma cells have not yet been explored.

In this study, the in vitro and in vivo antisynovial sarcoma activity of epi-1 and its underlying mechanisms were examined. Epi-1 showed potent anticancer activity in human synovial sarcoma SW982 cells and in a SW982 xenograft model. Mechanistically, epi-1 increased intracellular calcium levels, which stimulated reactive oxygen species (ROS) production and calpain activity, subsequently causing mitochondrial damage and cell death. Interestingly, extracellular signal-regulated kinase (ERK) was also induced by epi-1 treatment and seems to play a role in protecting against epi-1-induced cytotoxicity.

## 2. Results

### 2.1. Epi-1 Induction of Cytotoxicity in Synovial Sarcoma Cells is Dependent on its Folding Structure

**Figure 1:**
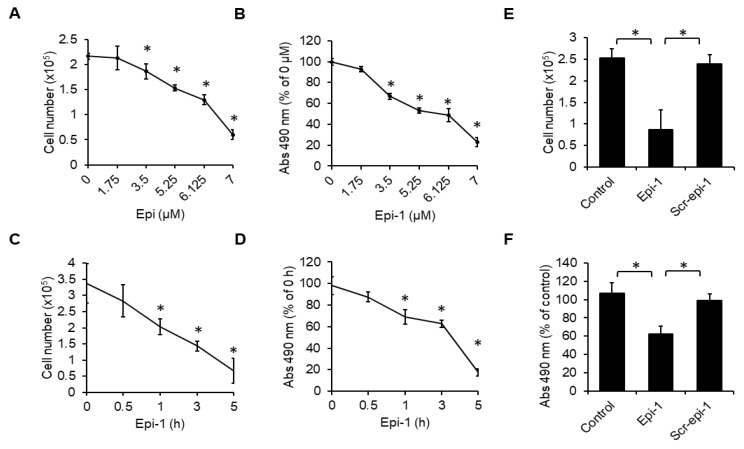
Cytotoxicity of epinecidin-1 (epi-1) on human synovial sarcoma cells. SW982 cells were treated with indicated doses of epi-1 for 5 h. Cytotoxicity was analyzed by the trypan blue exclusion assay (**A**) and MTS/PMS assay (**B**). Cells were treated with epi-1 (6.125 μM) at different time points (0.5, 1, 3, 5 h). Cytotoxicity was analyzed by the trypan blue exclusion assay (**C**) and MTS/PMS assay (**D**). Cells were treated with epi-1 (6.125 μM) or scrambled epi-1 (scr-epi-1) (6.125 μM) for 5 h, and cytotoxicity was determined by the trypan blue exclusion assay (**E**) and MTS/PMS assay (**F**). * *p* < 0.05 was considered significant.

To test the cytotoxicity of epi-1 in human synovial sarcoma cells, the SW982 cell line was treated with a range of epi-1 doses (0, 1.75, 3.5, 5.25, 6.125, and 7 μM). Cytotoxicity was determined by counting viable cells (Figure 1A) and the MTS/PMS assay (Figure 1B). The results show that cell death was induced by epi-1 in a dose-dependent manner. To determine the time course of epi-1-induced death, cells were treated with epi-1 and examined at various time points (0, 0.5, 1, 3, and 5 h). We found that epi-1-induced loss of cell viability was statistically significant beginning at 1 h after treatment (Figure 1C,D). To determine whether the α-helical structure is essential for epi-1-mediated cytotoxicity in synovial sarcoma cells, SW982 cells were treated with epi-1 or scr-epi-1 (scrambled epi-1; has the same charge as epi-1, but its secondary structure is an imperfect α-helix) [13]. Again, cytotoxicity was determined by viable cell count (Figure 1E) and the MTS/PMS assay (Figure 1F). Scr-epi-1 failed to induce cytotoxicity, suggesting that α-helical folding is required for epi-1-mediated cytotoxicity in SW982 cells.

### 2.2. Epi-1 Triggers Caspase-Independent Cell Death in SW982 Cells

**Figure 2:**
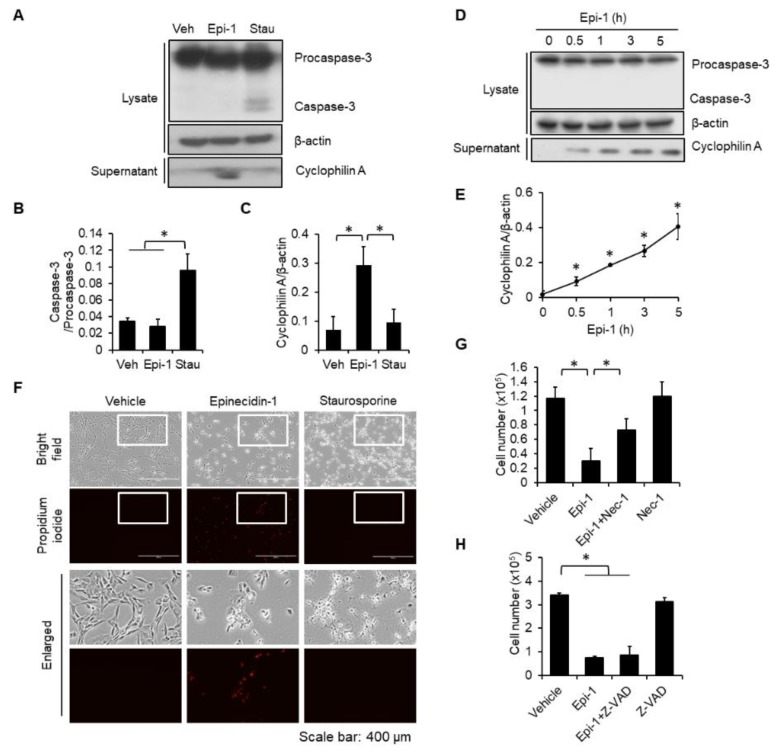
Epi-1 triggers caspase-independent cell death. (**A**,**C**) Cells were treated with epi-1 (6.125 μM) or stau (1 μM) for 3 h. Supernatants were collected and immunoblotted for cyclophilin A. Cell lysates were collected and immunoblotted for caspase-3 and β-actin. (**A**,**B**) Band intensities were quantified by ImageJ. (**D**) Cells were treated with epi-1 for different times, and cell lysates and supernatants were collected and immunoblotted with indicated antibodies. (**E**) Band intensities were quantified. (**F**) Cells were treated with epi-1 or stau as described in (**A**). After stimulation, cells were loaded with propidium iodide (PI; 1 μg/mL) for 10 min. After rinsing cells with PBS, PI incorporation was observed by fluorescence microscopy. Cells were pretreated with Necrostatin-1 (Nec-1) (10 μM) (**G**) or Z-VAD-FMK (Z-VAD) (100 μM) (**H**) for 1 h, followed by epi-1 (6.125 μM) treatment for 24 h. Cytotoxicity was determined by the trypan blue exclusion assay. * *p* < 0.05 was considered significant.

Various cell death pathways can be induced by antimicrobial peptides, such as apoptosis and necrosis [15,16]. To determine which cell death pathway is involved in epi-1-mediated death of synovial sarcoma cells, whole-cell lysates were collected from epi-1- and staurosporine (stau; apoptosis inducer)-treated SW982 cells, followed by immunoblotting with a caspase-3 antibody. Activation of caspase-3 was induced by stau but not epi-1 (Figure 2A,B). To monitor whether apoptosis may occur at an earlier time, cells were tested at various time points after treatment (0.5, 1, 3, and 5 h). Consistently, epi-1 did not induce activation of caspase-3 at any tested time point (Figure 2D). Next, the involvement of necrosis in epi-1-mediated cell death was examined. Extracellular cyclophilin A is considered to be a marker of necrosis [17], and epi-1 effectively increased the levels of cyclophilin A in the culture supernatant (Figure 2A,C–E). In contrast, extracellular cyclophilin A was not increased by stau (Figure 2A,C). Epi-1-treated cells also exhibited propidium iodide incorporation, while stau-treated cells did not (Figure 2F). Furthermore, the necrosis inhibitor, Necrostatin-1 (Nec-1), suppressed epi-1-induced toxicity (Figure 2G), but apoptosis inhibitor Z-VAD-FMK (Z-VAD) did not (Figure 2H).

### 2.3. Calcium and Calpain are Required for Epi-1-Induced Cell Death

**Figure 3:**
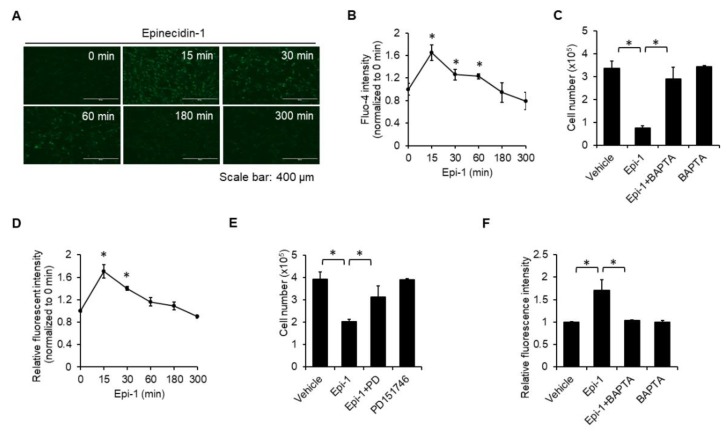
Calcium-dependent calpain activation is required for epi-1-mediated cytotoxicity. Cells were preloaded with Fluo-4 (5 μM) for 15 min, then treated with epi-1 at different points as indicated. Fluorescence of Fluo-4 was observed by fluorescence microscopy (**A**) and flow cytometry (**B**). (**C**) Cells were preincubated with BAPTA (BA; 10 μM) for 1 h, followed by epi-1 for an additional 5 h. Cytotoxicity was assessed by the trypan blue exclusion assay. (**D**) Cells were preloaded with fluorogenic calpain substrate t-BOC (10 μM) for 30 min, followed by epi-1 for the indicated times. (**E**) Cells were preincubated with PD151746 (PD) for 1 h, followed by epi-1 for an additional 5 h. Cytotoxicity was determined by the trypan blue exclusion assay. (**F**) Cells were pretreated with BA (10 μM) for 1 h, followed by epi-1 for an additional 15 min. Calpain activity was assessed. * *p* < 0.05 was considered significant.

Necrosis often involves intracellular calcium overload, which subsequently activates cell death-inducing molecules, such as calpain [18]. Epi-1 treatment elevated the intracellular calcium level within 15 min, and the elevation was sustained to 60 min (Figure 3A,B). Calcium chelator BAPTA blocked cell death, suggesting that calcium is necessary for epi-1-mediated cytotoxicity (Figure 3C). Calpain activity was also quickly induced within 15 min (Figure 3D), and suppression of calpain activity by PD151746 inhibited epi-1-mediated cytotoxicity (Figure 3E). Since BAPTA attenuated epi-1-mediated upregulation of calpain activity (Figure 3F), calcium seems to be required for epi-1-mediated activation of calpain.

### 2.4. Epi-1 Induces Mitochondrial Hyperpolarization

**Figure 4:**
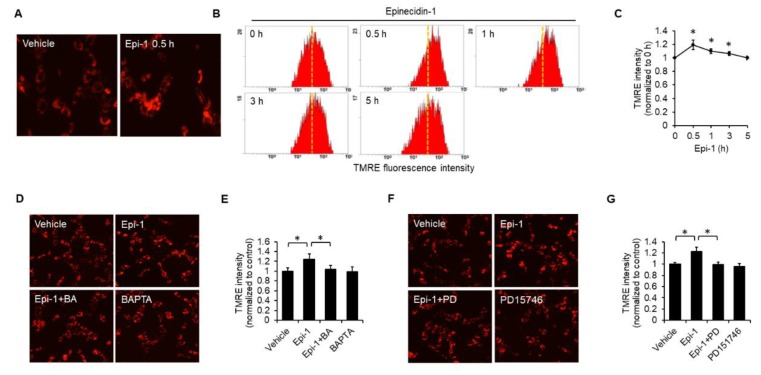
Calcium-dependent calpain activation plays an essential role in epi-1-induced mitochondrial hyperpolarization. Cells were treated with epi-1 for the indicated times, followed by incubation with TMRE (100 nM) for 15 min. Fluorescence intensity of TMRE was assessed by fluorescence microscopy (**A**) and flow cytometry (**B**,**C**). Dotted line: Basal TMRE levels. Cells were pretreated with BAPTA (10 μM) for 1 h, followed by epi-1 for an additional 0.5 h. TMRE intensity was assessed by fluorescence microscopy (**D**) and flow cytometry (**E**). Cells were preincubated with PD151746 (PD) for 1 h, followed by epi-1 for an additional 0.5 h. TMRE intensity was assessed by fluorescence microscopy (**F**) and flow cytometry (**G**). All fluorescent microscope images were taken under in 20× magnification. * *p* < 0.05 was considered significant.

Next, we analyzed the effect of epi-1 on mitochondrial function by TMRE. We found that epi-1-triggered mitochondrial hyperpolarization occurs within 30 min and is sustained to 3 h (Figure 4A–C). Both BAPTA (Figure 4D,E) and PD151746 (Figure 4F,G) suppressed epi-1-induced mitochondrial hyperpolarization, suggesting that calcium induction of calpain is required for epi-1 to cause mitochondrial hyperpolarization.

### 2.5. Epi-1 Induces Oxidative Stress and Downregulation of Antioxidant Proteins

**Figure 5:**
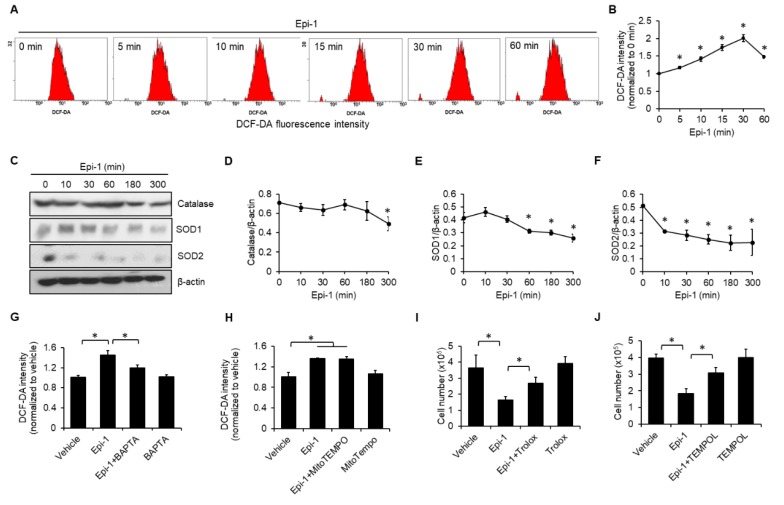
Epi-1 induces reactive oxygen species (ROS) generation and reduces expression of antioxidant proteins. (**A**,**B**) Cells were treated with epi-1 for different times, as indicated. ROS levels were monitored by DCF-DA (10 μM) using flow cytometry. (**C**) Cells were treated with epi-1 as described in (**A**), cell lysates were collected and immunoblotted with anti-catalase, anti-SOD1, anti-SOD2, and anti-β-actin antibodies. (**D**–**F**) Band intensities were measured with ImageJ. Cells were preincubated with BAPTA (10 μM) (**G**) and MitoTEMPO (10 μM) (**H**) for 1 h, followed by epi-1 for an additional 30 min. Fluorescence intensity of DCF-DA was assessed by flow cytometry. Cells were preincubated with Trolox (100 μM) (**I**) and TEMPOL (150 μM) (**J**) for 1 h, followed by epi-1 for an additional 5 h. Cytotoxicity was scored by the trypan blue exclusion assay. * *p* < 0.05 was considered significant.

Intracellular calcium overload has been linked to increased reactive oxygen species (ROS) [19], so we also monitored intracellular ROS. After epi-1 treatment, ROS generation was elevated within 5 min and reached a peak at 30 min (Figure 5A,B). Next, we determined the effect of epi-1 on the abundance of antioxidant proteins, including catalase, superoxide dismutase 1 (SOD1), and superoxide dismutase 2 (SOD2). The levels of catalase (Figure 5C,D), SOD1 (Figure 5C,E), and SOD2 (Figure 5C,E) were all decreased by epi-1. Moreover, BAPTA reduced ROS generation after epi-1 treatment, suggesting that calcium acts upstream of ROS (Figure 5G). In contrast, MitoTEMPO did not suppress epi-1-induced ROS generation (Figure 5H). Since ROS scavengers Trolox (Figure 5I) and TEMPOL (Figure 5J) both attenuated epi-1-induced cytotoxicity, we conclude ROS plays a role in toxicity. Together, these results demonstrate that epi-1 induces excessive ROS generation and downregulation of antioxidant proteins. Furthermore, calcium-dependent ROS generation is important for epi-1-mediated cytotoxicity.

### 2.6. ERK Plays a Protective Role in Epi-1-Mediated Cell Death

**Figure 6:**
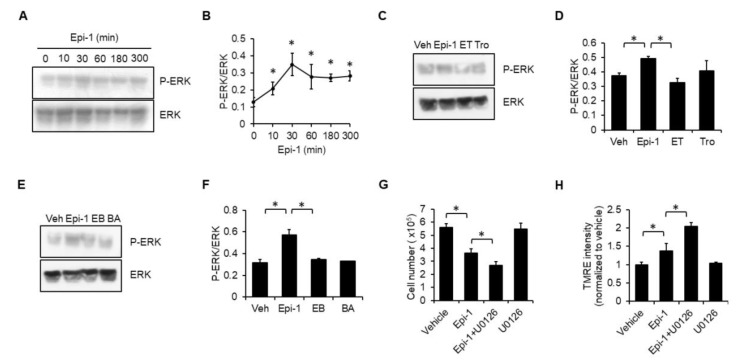
Activation of extracellular signal-regulated kinase (ERK) signaling plays a protective role in epi-1-induced cytotoxicity. (**A**) Cells were treated with epi-1 for different times, as indicated. Whole cell lysates were collected and immunoblotted with anti-phospho-ERK and anti-ERK antibodies. (**B**) Band intensities were measured with ImageJ. (**C**) Cells were preincubated with Trolox (Tro; 100 μM) for 1 h, followed by epi-1 for an additional 0.5 h. ERK activation was determined as described in (A). Veh: vehicle; ET: epi-1+Trolox; Tro: Trolox. (**D**) Band intensity was measured with ImageJ. (**E**) Cells were preincubated with BAPTA (BA; 10 μM) for 1 h, followed by epi-1 for an additional 0.5 h. ERK activation was determined as described in (A). (**F**) Band intensity was measured with ImageJ. Veh: vehicle; EB: epi-1+BAPAT; BA: BAPTA. (**G**) Cells were preincubated with U0126 (20 μM) for 1 h, followed by epi-1 for an additional 5 h. Cytotoxicity was determined by the trypan blue exclusion assay. (**H**) Cells were preincubated with U0126 for 1 h, followed by epi-1 for an additional 30 min. TMRE intensity was analyzed by flow cytometry. * *p* < 0.05 was considered significant.

ERK has been considered as a therapeutic target of soft tissue sarcoma due to it is associations with cell proliferation, differentiation, and survival [20]. We found that ERK phosphorylation is stimulated by epi-1 treatment (Figure 6A,B). Both Trolox (Figure 6C,D) and BAPTA (Figure 6E,F) effectively inhibited epi-1-induced ERK phosphorylation, and the ERK inhibitor, U0126, potentiated epi-1-mediated cytotoxicity (Figure 6G) and mitochondrial hyperpolarization (Figure 6H).

### 2.7. In Vivo Antisynovial Sarcoma Ability of Epi-1

**Figure 7:**
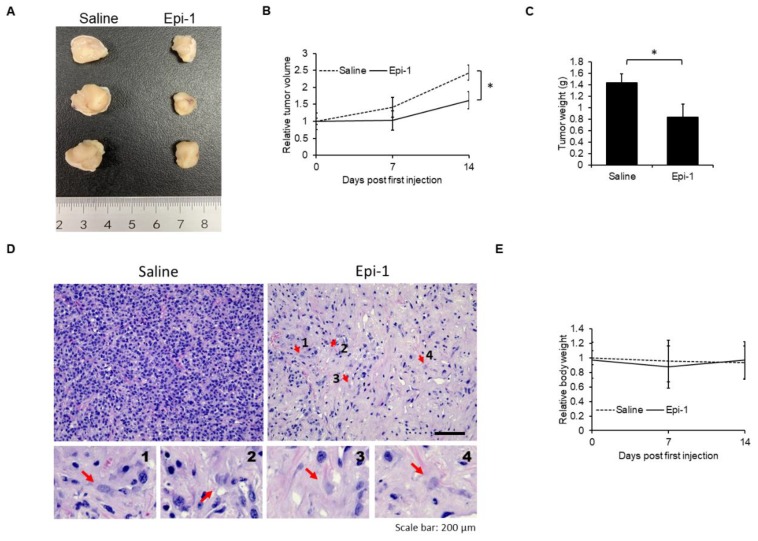
Effect of epi-1 on a subcutaneous synovial sarcoma mouse xenograft model. Xenograft tumor size (length unit: centimeter) (**A**), relative tumor volume (**B**), tumor weight (**C**), H&E analysis of tumors (**D**), and relative body weight (**E**) after epi-1 or saline treatment. Red arrows indicate karyolytic cells. Magnified pictures of indicated karyolytic cells are shown in the lower images. Scale bar: 200 μm. Significance was determined by one-way ANOVA. * *p* < 0.05 was considered significant.

To determine efficacy of epi-1 against antisynovial sarcoma in vivo, epi-1 and/or saline were injected into tumor xenografts every two days. Epi-1-treated tumors exhibited smaller tumor size, reduced tumor volume, and lower tumor weight than saline-treated controls (Figure 7A–C). H&E histological analysis demonstrated that epi-1-treated tumors also exhibited a reduced number of nuclei compared to saline-treated tumors (Figure 7D). Furthermore, the number of karyolytic cells, a hallmark of necrosis [21], was significantly increased in the epi-1-treated samples (indicated by red arrows in Figure 7D). Importantly, epi-1 treatment did not affect the body weight of the mice, suggesting that the treatment did not cause overt negative systemic effects (Figure 7E). Together, these results demonstrated that epi-1 possesses antisynovial sarcoma activity in vivo.

## 3. Discussion

Excessive calcium induces calpain activation, which has been linked to lysosomal and mitochondrial permeabilization [22,23]. As such, calpain is known to cleave membrane proteins required for the structural integrity of the lysosome, including lysosome-associated membrane protein 2 [23]. Similarly, calpain also cleaves Bid to form tBid, which causes mitochondrial permeabilization [24]. Damage to either mitochondria or lysosomes will release intra-organelle proteases that cause cell death [25,26]. In addition, organelle damage usually leads to excessive ROS generation and oxidative stress [27], which also contributes to cell death. In addition to these mechanisms of cell death, calpain activation is known to initiate α2β1 integrin degradation by endosomes [28]. Because α2β1 integrin plays an essential role in promoting migration and invasion in human osteosarcoma cell lines [29], the migration and invasion activities of cancer cells might be especially susceptible to epi-1 treatment and its induction of calpain activation.

Conventional chemotherapeutic agents may be suboptimal cancer treatment options, partially because they commonly induce apoptosis as the major cell death pathway [30]. Unfortunately, apoptotic machinery is often defective in cancer cells, which may explain many cases of cancer chemotherapy failure [31]. Furthermore, chemotherapy-induced adverse effects are usually the main dose-limiting factors and reason for discontinuation of therapy [32]. Conversely, antimicrobial peptides possess several advantages as potential anticancer drugs. For example, antimicrobial peptides are cationic short-chain peptides that are readily attracted to cancer cells by virtue of a membrane that is negatively charged relative to that of noncancerous cells [33]. This property allows for selective killing of cancer cells by antimicrobial peptides. Exposure up to 11 μM did not cause cytotoxicity on human immortalized keratinocyte cell line HaCaT [34]. Additionally, epi-1 induces synovial sarcoma cell death by necrosis. Induction of necrosis may be an effective approach to eliminate apoptosis-defective cancer cells [35]. Furthermore, release of cyclophilin A is stimulated by epi-1-induced necrosis, and cyclophilin A-mediated macrophage differentiation, migration, and proliferation may contribute to tumor suppression [36]. Notably, epi-1 exerts anticancer effects against various types of cancers by different mechanisms. For example, epi-1 induces lytic cell death in fibrosarcoma [14], but it triggers cells death through apoptosis in U937 human leukemia cells [37]. In this study, we found that epi-1 induces necrosis in human synovial sarcoma cells. Thus, epi-1 may initiate cell death through different pathways in a cell-type-dependent manner, and it may be especially useful as an anticancer agent in synovial sarcoma.

The rapid tumor suppression activity and induction of necrosis endow epi-1 with high potential and promise as an anticancer agent for apoptosis-defective and chemoresistant cancer cells in human synovial sarcoma. Although current formulations of epi-1 may not be suitable for intravenous use due to protease sensitivity, intratumoral injection of epi-1 could be considered for reducing tumor size before resection.

## 4. Materials and Methods

### 4.1. Reagents

Epi-1 (H-GFIFHIIKGLFHAGKMIHGLV-OH) was synthesized by GL Biochem (Shanghai, China). Epi-1 was dissolved in normal saline. Propidium iodide (PI), 2′,7′-dichlordihydrofluorescein diacetate (DCF-DA), Trolox, TEMPOL, MitoTEMPO, BAPTA (BA), U0126, PD151746, Necrostatin-1 (Nec-1), staurosporine (stau), and DMSO were purchased from Sigma (Merck KGaA, Darmstadt, Germany). MTS and PMS were purchased from Promega (Madison, WI, USA). Trypan blue, Fluo-4, AM (Fluo-4), tetramethylrhodamine, ethyl ester (TMRE), and *t*-BOC-Leu-Met-CMAC (*t*-BOC) were purchased from Thermo Fisher Scientific (Waltham, MA, USA). Z-VAD-FMK was purchased from Cell Signaling (Danvers, MA, USA).

### 4.2. Cell Culture

Human synovial sarcoma cell line SW982 was purchased from the Bioresource Collection and Research Center (Hsinchu, Taiwan). Cells were maintained in Dulbecco’s Modified Eagle’s medium (DMEM; Gibco, ThermoFisher, Waltham, MA, USA), supplemented with 10% fetal bovine serum (FBS; Gibco), 2 mM L-glutamine, and antibiotic-antimycotic (FBS; Gibco).

### 4.3. Cytotoxic Assay

Cytotoxicity was determined as previously described [15,38]. Briefly, after stimulation, culture supernatant and attached cells were collected using trypsin, and viable cell count was calculated by trypan blue exclusion assay. For MTS/PMS assay, MTS/PMS mixed reagent was added to cells after treatment and was incubated at 37 °C for 20 min. The absorbance at OD490 nm was recorded using an ELISA plate reader. For the PI exclusion assay, cells were treated with the indicated treatment followed by PI for 10 min. Thereafter, cells were rinsed with PBS and observed under fluorescence microscopy (EVOS FL Cell Imaging System, ThermoFisher, Waltham, MA, USA).

### 4.4. Western Blotting

After treatment, supernatants were collected in 6X sample buffer (Sigma). Cell lysates were collected in RIPA buffer (Merck Millipore, Burlington, MA, USA). Thereafter, supernatants and cell lysates were separated by SDS-PAGE and transblotted onto PVDF membrane (GE Healthcare Life sciences, Pittsburgh, PA, USA). Target protein abundance was detected using the indicated antibodies. Equal amounts of protein were loaded for each sample. For detection of caspase-3 and β-actin, blots were first probed with caspase-3 antibody, followed by stripping and re-probing with β-actin. For detection of antioxidant proteins, the membrane was cut into three sections according to the molecular weight for each target protein. The cut blots were probed for catalase, SOD1/SOD2, and β-actin antibodies. Because the molecular weights of SOD1 and SOD2 are very close, we probed one membrane section for SOD1 first, followed by stripping and re-probing for SOD2. Molecular weight marker was purchased from ThermoFisher (Waltham, MA, USA). Band intensity was measured by ImageJ software (1.51j8; NIH, Bethesda, MD, USA). All antibodies used in this study were purchased from Cell Signaling.

### 4.5. ROS Measurement

ROS was monitored using DCF-DA by flow cytometry. Briefly, cells were preincubated with DCF-DA (10 μM) for 10 min, followed by epi-1 stimulation. Cells were then rinsed with PBS. Fluorescence intensity of DCF-DA was assessed by flow cytometry (Beckman Coulter, Indianapolis, IN, USA).

### 4.6. Calcium and Calpain Activity

To determine whether epi-1 modulates intracellular calcium levels, cells were preincubated with Fluo-4 (5 μM) for 30 min, followed by epi-1 treatment. Cells were then rinsed with PBS. Fluorescence intensity of Fluo-4 was observed by microscopy and flow cytometry. Calpain activity was monitored with a cell-permeable fluorogenic calpain substrate *t*-BOC [39]. Briefly, cells were preloaded with *t*-BOC (20 μM) for 1 h, followed by epi-1 treatment. After stimulation, cells were rinsed with PBS, and fluorescence intensity of *t*-BOC was assessed by fluorescence microscopy and flow cytometry (Beckman Coulter).

### 4.7. Mitochondrial Function

Mitochondrial function was determined as previously described [15,38]. Briefly, cells were preloaded with TMRE (100 nM) for 15 min, after which the excess TMRE was washed off with PBS. Fluorescence intensity was monitored by fluorescence microscopy and flow cytometry (Beckman Coulter).

### 4.8. Human Synovial Sarcoma Xenograft Nude Mice Model

Mouse experiments were approved by the Academia Sinica Institutional Animal Care & Utilization Committee (Protocol number: IACUC 16-06-973). One-month-old male nude mice (NU/NU) were purchased from BioLASCO (Taipei, Taiwan). Mice were allowed to adapt to the environment for 2 weeks before experimentation. To establish a synovial sarcoma model in nude mice, experimental procedures followed a previous study with minor modifications [40]. SW982 cells (4 × 10^6^ cells in 50 μL PBS and 50 μL Matrigel matrix) were subcutaneously inoculated into nude mice to form tumor xenografts. When tumor size reached 120–180 mm^3^, mice were randomly assigned into two groups (*n* = 3 for each group), saline and epi-1 (250 μg in 100 μL saline). Saline or epi-1 were administered by intratumor injection once every two days for a total of seven injections. Tumor size and volume were measured as previously described [41]. Tumors samples were harvested at day 14 for H&E histological analysis.

### 4.9. Statistical Analysis

In vitro experiments were performed in triplicate with at least three independent replicates. Results from in vitro and in vivo experiments were analyzed by one-way ANOVA using GraphPad Prism 5.0 software. *p* < 0.05 was considered significant.

## 5. Conclusions

**Figure 8:**
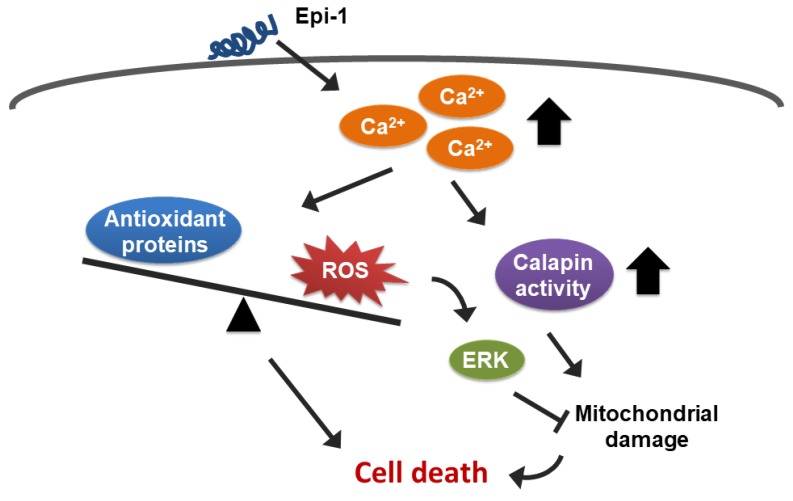
A summary diagram showing how epi-1 induces cell death in synovial sarcoma cells. Epi-1 increases intracellular levels of calcium. Elevated calcium induces oxidative stress and calpain activity. Activated calpain causes mitochondrial damage. Both mitochondrial damage and oxidative stress contribute to cell death. ROS also activates ERK, which plays a protective role in epi-mediated cytotoxicity. Thin arrowheads: induction; Thick arrowheads: elevation; T bar: inhibition.

In this report, we demonstrate that epi-1 exerts antitumor activity in synovial sarcoma cells in vitro and in vivo. Furthermore, the mechanism underlying epi-1 toxicity involves increased intracellular calcium and subsequent calpain activation. Importantly, activation of calpain is required for epi-1-mediated mitochondrial dysfunction and cell death. In addition, elevated calcium induces ROS generation and diminishes the levels of antioxidant proteins, which also contributes to cell death in human synovial sarcoma cells (Figure 8).
